# Genome-Wide Progesterone Receptor Binding: Cell Type-Specific and Shared Mechanisms in T47D Breast Cancer Cells and Primary Leiomyoma Cells

**DOI:** 10.1371/journal.pone.0029021

**Published:** 2012-01-17

**Authors:** Ping Yin, Damian Roqueiro, Lei Huang, Jonas K. Owen, Anna Xie, Antonia Navarro, Diana Monsivais, John S. Coon V, J. Julie Kim, Yang Dai, Serdar E. Bulun

**Affiliations:** 1 Division of Reproductive Biology Research, Department of Obstetrics and Gynecology, Feinberg School of Medicine, Northwestern University, Chicago, Illinois, United States of America; 2 Department of Bioengineering, University of Illinois at Chicago, Chicago, Illinois, United States of America; National Cancer Center, Japan

## Abstract

**Background:**

Progesterone, via its nuclear receptor (PR), exerts an overall tumorigenic effect on both uterine fibroid (leiomyoma) and breast cancer tissues, whereas the antiprogestin RU486 inhibits growth of these tissues through an unknown mechanism. Here, we determined the interaction between common or cell-specific genome-wide binding sites of PR and mRNA expression in RU486-treated uterine leiomyoma and breast cancer cells.

**Principal Findings:**

ChIP-sequencing revealed 31,457 and 7,034 PR-binding sites in breast cancer and uterine leiomyoma cells, respectively; 1,035 sites overlapped in both cell types. Based on the chromatin-PR interaction in both cell types, we statistically refined the consensus progesterone response element to G•ACA• • •TGT•C. We identified two striking differences between uterine leiomyoma and breast cancer cells. First, the *cis*-regulatory elements for HSF, TEF-1, and C/EBPα and β were statistically enriched at genomic RU486/PR-targets in uterine leiomyoma, whereas E2F, FOXO1, FOXA1, and FOXF sites were preferentially enriched in breast cancer cells. Second, 51.5% of RU486-regulated genes in breast cancer cells but only 6.6% of RU486-regulated genes in uterine leiomyoma cells contained a PR-binding site within 5 kb from their transcription start sites (TSSs), whereas 75.4% of RU486-regulated genes contained a PR-binding site farther than 50 kb from their TSSs in uterine leiomyoma cells. RU486 regulated only seven mRNAs in both cell types. Among these, adipophilin (PLIN2), a pro-differentiation gene, was induced via RU486 and PR via the same regulatory region in both cell types.

**Conclusions:**

Our studies have identified molecular components in a RU486/PR-controlled gene network involved in the regulation of cell growth, cell migration, and extracellular matrix function. Tissue-specific and common patterns of genome-wide PR binding and gene regulation may determine the therapeutic effects of antiprogestins in uterine fibroids and breast cancer.

## Introduction

The steroid hormone progesterone not only plays a central role in the development, growth, and differentiation of the female reproductive system, but also is involved in the development and progression of reproductive diseases, including endometriosis, endometrial cancer, ovarian cancer, breast cancer, and uterine leiomyoma [1], [2], [3], [4], [5], [6]. Studies suggest that breast cancer and uterine leiomyoma may share some similarities with regard to the function of progesterone in growth regulation of these tissues [7], [8], [9]; however, the effect of progesterone on increasing the risk for breast cancer and leiomyoma continues to be debated.

Various experimental models have provided seemingly conflicting results. For example, uterine leiomyoma growth is stimulated by estrogen but not by progesterone in the *in vivo* Eker rat model [10]. Yet *in vitro*, progestin shows similar growth-promoting effects as estrogen on primary cultures of human uterine leiomyoma cells, by inhibiting apoptosis and stimulating proliferation [11], [12], [13]. Most importantly, in an *in vivo* human leiomyoma xenograft model, progesterone and its receptor (PR) directly stimulated growth, whereas the key action of estrogen and its receptor was to maintain PR expression in leiomyoma tissue [14].

Clinical evidence has suggested that progesterone plays a critical role in the growth of leiomyoma. Proliferation markers such as Ki67 and proliferating cell nuclear antigen (PCNA) are highest in leiomyoma in the luteal/secretory phase [15], [16], [17]. Furthermore, quantitative proliferation indices of leiomyoma in postmenopausal women increase significantly with combined estrogen plus progestin replacement but not with estrogen replacement alone [15]. Most importantly, antiprogestins such as RU486 (mifepristone) and J867 (asoprisnil) reduce the size of uterine fibroids [18], [19].

In breast cancer, both positive and negative effects of progestins on breast cancer progression have been recapitulated in cellular and animal models of this disease [20]. In T47D cells, for example, progestins induce the expression of E2F1 and cyclin D1, facilitate hyperphosphorylation of Rb, and initiate cell replication [21], [22]. When the same cells are implanted in athymic nude mice, robust progestin-dependent tumor growth is observed [23]. In other cell lines, such as MCF-7, progestins efficiently inhibit estrogen-dependent cell proliferation *in vitro*, an activity that has generally been considered to underlie their efficacy as breast cancer therapeutics [24].

The antiprogestin RU486 has been shown to prevent mammary tumorigenesis in mice deficient in tumor suppressors [25]. Clinically, high-dose progestins are occasionally administered to women with advanced breast cancer to enhance their overall well-being. In these cases, however, high-dose progestin is not intended to produce a specific therapeutic effect on breast cancer tissue. The most definitive evidence that progesterone promotes breast cancer came from the Women's Health Initiative Study. Breast cancer incidence significantly increased in women assigned to the estrogen plus progestin arm, whereas the risk was surprisingly lower in those who were taking estrogens alone [26].

These data, combined with known expression patterns of estrogen receptor alpha (ERα) and PR in breast tumors, suggest a permissive role of estrogen/ERα, through induction of PR expression in benign breast tissue or tumors. PR that is liganded by progesterone or a progestin seems to be the downstream trigger of tumorigenesis in both uterine leiomyoma and breast cancer [14], [18], [25], [27], [28]. Taken together, this body of evidence highlights the need to define the roles of progestins, antiprogestins, and their cognate receptors in reproductive diseases including breast cancer and leiomyoma, as a first step in the development of strategies to optimally exploit this signaling axis for the identification of clinically useful pharmaceuticals.

The genomic activity of progesterone is to a large extent mediated by the two PR isoforms, PR-A and PR-B, which belong to the nuclear receptor superfamily of ligand-regulated transcription factors (TFs; for a review, see reference [29]). Classically, PR regulates expression of target genes by binding directly to its cognate sequence, the progesterone response element (PRE). Alternatively, nontraditional regulation involves protein-protein interactions with other DNA-binding proteins such as SP1 and AP1 [30], [31]. Identification of the PR target gene network regulated by agonists and/or antagonists is essential to gain a clearer understanding of the role of PR in reproductive tissue disorders.

Recently, high-throughput ER chromatin immunoprecipitation sequencing (ChIP-seq) successfully identified a large number of ER-binding sites in breast cancer cells [30], [32], [33], which has transformed our understanding of how ER acts to alter cellular function. To date, no similar information is available regarding the genome-wide binding pattern of PR to its downstream genes.

Selective progesterone receptor modulators (SPRM) are partial progesterone antagonists used for the treatment of breast cancer and uterine leiomyoma. One of the most widely used is RU486, which has mixed agonistic/antagonistic properties and tissue-specific effects [34], [35]. SPRMs may have a higher affinity for PR and induce an alternative conformation of the PR ligand-binding domain that results in the recruitment of different co-factors and regulation of gene transcription [36], [37], [38]. From a translational perspective, the effect of a SPRM on genome-wide PR binding and gene expression has not been studied to date.

Here, we took advantage of the observation that RU486 is therapeutic for two extremely common tumors of women—breast cancer and uterine leiomyoma—and used massive parallel sequencing of immunoprecipitated DNA fragments to identify PR-interaction sites in response to RU486. We coupled this approach with gene expression profiling to determine common and tissue-specific mechanisms of RU486 action in both cell types.

## Results

### Genome-wide binding landscape of PR in breast cancer T47D and uterine leiomyoma cells

**Figure 1:**
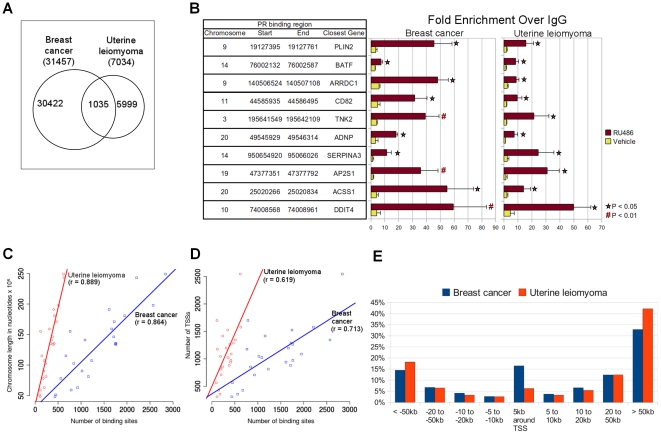
Summary of PR-binding sites in uterine leiomyoma and breast cancer cells. (A) Venn diagrams summarize the number of PR-binding sites in T47D breast cancer cells and uterine leiomyoma cells. (B) Validation of *in vivo* recruitment of PR to novel PR-binding regions identified by ChIP-seq. T47D and leiomyoma cells (from 5 different subjects) were treated with RU486 (10^−6^ M) or vehicle (ethanol) for 1 hour. ChIP was performed using anti-PR or nonspecific rabbit IgG, followed by real-time PCR. Data presented are expressed as fold enrichment of PR relative to IgG, and are the average of three to five independent ChIP experiments. (C) Correlation between the number of PR-binding sites in an individual chromosome (X-axis) to the chromosome length (Y-axis). (D) Correlation between the number of PR-binding sites (X-axis) with the number of TSSs (Y-axis) in an individual chromosome. (E) Distribution of PR-binding sites relative to their nearest TSSs. The percentages of all identified PR-binding sites in each cell type and region are shown.

We performed PR ChIP followed by deep sequencing using breast cancer T47D and human uterine leiomyoma cells that had been treated with RU486 for 1 hour. We identified 31,457 unique PR-binding sites in breast cancer cells and 7,034 in leiomyoma cells. A total of 1,035 binding sites were common to both cell types, i.e., their locations overlapped (with an average overlap length of 500 bp) (Figure 1A). ChIP-quantitative PCR analysis validated the binding of PR to ten representative ChIP-seq peaks with full or half PREs common to T47D and leiomyoma cells (Figure 1B). We showed that treatment with RU486 (*vs.* vehicle ethanol) significantly enhanced binding of PR to these sites. The range of RU486-dependent fold-enrichment for ChIP-derived DNA was 7.1 to 59.4 in T47D cells and 7.3 to 49.8 in leiomyoma cells. The PR-binding sites in the vicinity of the genes, PLIN2, BATF, ARRDC1, SERPINA3, and AP2S1, contained half PRE motifs, whereas those associated with CD82, TNK2, ADNP, ACSS1, and DDIT4 contained full PRE motifs. These results suggested that these novel sites are bona fide and functional PR-binding sites. Furthermore, in comparison with the recently published 18 PR-binding sites in leiomyoma cells, 12 out of those 18 (66.7%) binding sites were recovered in the present study including those close to the genes, KLF11, SLC7A8, SLC18A2, FLT4, RABGGTA, MAN1C1, PLCH2, TRPV3, AP3D1, LMAN1L, PRDM16 and KIAA1267 [7]. Furthermore, the current ChIP-seq analysis also confirmed PR binding to a number of previously known PR-target genes such as MUC1, FKBP5 and E2F1 [22], [39], [40].

We compared the locations of PR-binding sites relative to genomic annotations obtained from the UCSC RefSeq track of the Human Genome Assembly hg19 [41]. For both cell types, the number of binding sites per chromosome correlated strongly with chromosome length, with Pearson correlation coefficients of r = 0.864 for breast cancer cells and r = 0.889 for leiomyoma cells (p<1×10^−7^ for both cell types; Figure 1C). We also noted a correlation between the number of binding sites and the number of unique transcription start sites (TSSs) per chromosome: r = 0.713 (p<0.0001) for breast cancer cells and r = 0.619 (p<0.002) for leiomyoma cells (Figure 1D).

The distribution of the distances between PR-binding sites and the closest TSS to a known gene is shown in Figure 1E. Intriguingly, a higher number of PR-binding sites (5,174; 16.45% of all binding sites) lay 5 kb proximal (upstream or downstream) to a TSS in breast cancer cells compared with leiomyoma cells (442 sites; 6.28% of all binding sites). Within the 5 kb region upstream of a TSS, there was an approximately 3-fold higher likelihood (10.87% *vs.* 3.47%) of finding a PR-binding site in breast cancer *vs.* leiomyoma cells. In contrast, we found that 47.34% (14,892) of the PR-binding sites in breast cancer cells and 60.40% (4,249) in leiomyoma cells were located in regions farther than 50 kb from a TSS. In agreement with published data on nuclear receptor binding, 36.7% (breast cancer) and 40.4% (leiomyoma) of the binding sites were located within an intron [30], [42], [43]. In general, PR-binding activity was higher at regions downstream (*vs.* upstream) of a TSS, which may be partially due to the high intronic occupancy (Table S1). These findings suggest that DNA-bound PR may interact with the transcriptional machinery through both proximal- and distal-acting mechanisms.

### Transcription factor binding motif analysis of identified PR-binding sites

With the goal of identifying enriched TF binding motifs in all PR-binding sites, we scanned their sequences using position weight matrices (PWMs) from TRANSFAC [44]. For all PWMs from TRANSFAC, we ranked the enriched motifs in the PR-binding sites based on their enrichment p-values (See Materials and Methods; lower p-value means higher rank).

To examine similarities and differences, we assessed the ranking of enriched motifs (PWMs) and selected the first 100 motifs for each cell type. Because more than one PWM could map to the same TF, our results included the TF and its PWMs with statistically significant hits. The top 10 most common TFs represented by these subsets are listed in Table 1. Since the PRE motif is similar to that of the androgen response element (ARE) and glucocorticoid response element (GRE), it was not surprising to find that ARE and GRE were highly represented in this analysis. The remainder of the binding motifs included HOXA4; LHX2; Msx-1; Otx1,3; Pax-4,6; PITX1; Six-1,2,3,4,6; and SREBP-1. Among these, only Pax4 binding motifs were shown to be enriched in glucocorticoid receptor (GR)-binding sites, suggesting that although GR and PR share a similar binding motif, they may regulate unique sets of downstream target genes through interactions with specific TFs [45].

**Table 1:** Top 10 enriched TF binding motifs within PR-binding sites common to both T47D breast cancer cells and leiomyoma cells.

			Breast cancer		Leiomyoma	
Factor name	Description	TRANSFACmatrix ID	Z-value	p-value	Z-value	p-value
AR	Androgen receptor; NR3C4	V$AR_01	54.723	<1.0e-323	90.819	<1.0e-323
		V$AR_04	51.514	<1.0e-323	77.330	<1.0e-323
GR	Glucocorticoid receptor; NR3C1	V$GRE_C	36.067	1.3e-280	58.241	<1.0e-323
HOXA4	HOX1D	V$HOXA4_Q2	−44.989	<1.0e-323	−13.617	2.0e-038
LHX2	hLH-2; LH-2	V$LH2_01	−44.113	<1.0e-323	−12.073	8.3e-030
Msx-1	Msh homeobox 1; MSX1	V$MSX1_02	−55.425	<1.0e-323	−12.930	1.9e-034
Otx 1,3	Diencephalon/mesencephalon homeobox 1;	V$OTX1_01	−52.433	<1.0e-323	−18.657	9.7e-074
	Dmbx1	V$OTX3_01	−50.794	<1.0e-323	−17.122	8.2e-062
Pax- 4,6	Paired box genes 4 and 6; PAX4; PAX6	V$PAX4_02	−50.005	<1.0e-323	−18.592	3.3e-073
		V$PAX6_02	−41.860	<1.0e-323	−10.909	5.4e-024
PITX1	Pituitary homeobox 1; Ptx1	V$PITX1_01	−51.469	<1.0e-323	−20.052	1.8e-085
Six- 1,2,3,4,6	SIX homeobox 1, 2, 3, 4, and 6; Six1; Six3;	V$SIX1_01	−43.183	<1.0e-323	−14.400	3.5e-043
	Six6; Six9	V$SIX2_01	−41.374	<1.0e-323	−14.115	2.0e-041
		V$SIX3_01	−42.943	<1.0e-323	−14.693	4.9e-045
		V$SIX4_01	−38.304	<1.0e-323	−16.814	1.5e-059
		V$SIX6_01	−44.116	<1.0e-323	−13.853	7.9e-040
		V$SIX6_02	−41.634	<1.0e-323	−13.432	2.5e-037
SREBP-1	Sterol regulatory element binding protein 1	V$SREBP1_01	−38.048	<1.0e-323	−14.936	1.4e-046

We also identified a number of differences between breast cancer and leiomyoma cells with respect to enrichment of TF motifs at PR-binding sites. For example, binding motifs for E2F and Fox family members, including FOXO1 (FOXO1A), FOXA1 (HNF3A), and FOXF2 (FREAC2,4), are highly enriched in PR-binding sites in breast cancer cells but not in leiomyoma cells (Table S2). This is consistent with previous reports indicating that E2F binding is enriched in PR target genes, and that FOXA1 binding is enriched in ER and GR target genes [22], [45], [46]. On the other hand, we found that motifs for HSF, TEF-1, and C/EBPα and β were enriched in PR-binding sites in leiomyoma cells (Table S3). HSF binding has been previously reported to be enriched in GR targets, and C/EBP sites enriched in GR- and ERα-interaction sites [30], [45]. Interestingly, among the top 20 enriched TF binding motifs, we found that AP2 and SP1 were listed as the two most significantly enriched TFs in breast cancer cells, whereas AP1 was listed as the top enriched TF in leiomyoma cells; and SP1 and AP1 have been reported to be enriched proximal ER or GR targets in various cell types [30], [33], [47].

Finally, we found that three PREs listed in the TRANSFAC database were significantly enriched in both cell types (p<1×10^−12^, Table 2). In leiomyoma cells, 4.8% of PR targets contained the PR_01 and PR_02 motifs, representing complete PREs; the frequency of these motifs was approximately 5 times lower in breast cancer cells (1%). Strikingly, the enrichment of a third motif, which was a PRE half-site (PR_Q2), was markedly higher than either of the complete PREs in both breast cancer (28.7%) and leiomyoma cells (34.8%). These data suggest that PR interacts with half PREs at a higher frequency than with classical or complete PREs to regulate downstream target genes in these tissues.

**Table 2:** Enriched PREs at PR targets in T47D breast cancer cells and leiomyoma cells.

	Breast cancer		Leiomyoma		Common to the two cells	
Motif	Number of binding sites including the motif (%)	p-value	Number of binding sites including the motif (%)	p-value	Number of binding sites including the motif (%)	p-value
**PR_01**	108 (0.3%)	6.8e-017	124 (1.8%)	<1.0e-323	23 (2.2%)	3.0e-103
**PR_02**	204 (0.7%)	8.6e-049	208 (3.0%)	<1.0e-323	39 (3.8%)	<1.0e-323
**PR_Q2**	9,020 (28.7%)	1.7e-013	2,444 (34.8%)	1.7e-041	225 (21.7%)	3.6e-026

### Discovery of *de novo* motifs in PR-binding sites common to both cell types

In addition to our analyses guided by PWMs from TRANSFAC, we attempted to identify new motifs in PR targets common to both breast cancer and leiomyoma cells using MEME as the motif discovery tool [48]. We compared the discovered motifs to known motifs from the TRANSFAC and JASPAR databases [49].

**Figure 2:**
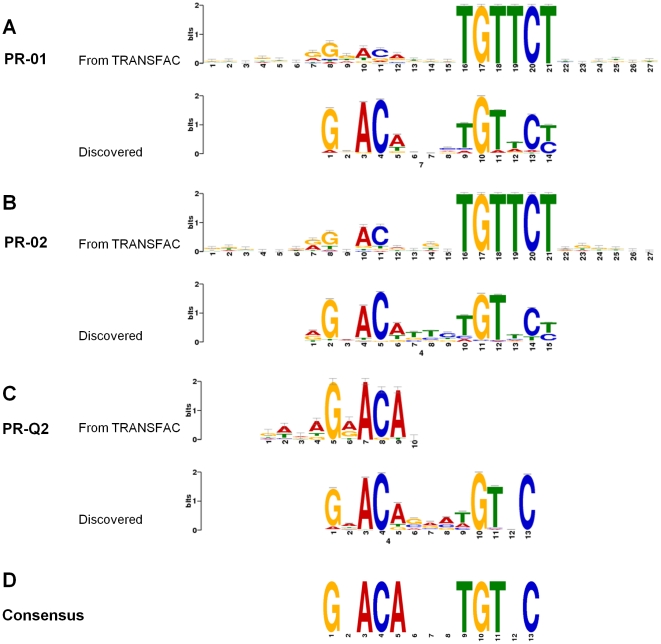
Sequence logos of PRE consensus motifs. The three known PRE motifs from TRANSFAC are shown in upper panels A (PR_01), B (PR_02), and C (PR_Q2). The consensus logo motifs derived using MEME analysis of ChIP-seq data from the 1,035 PR-binding sites common to both T47D breast cancer cells and leiomyoma cells are shown in lower panels of A, B, and C. Panel D shows the proposed consensus PRE motif based on the three TRANSFAC motifs and the discovered motifs from ChIP-seq data. For the consensus logos, the vertical axes (Bits) indicate the information content of the base frequency at that position. The horizontal axes refer to consensus site position.

Figure 2 shows the three discovered motifs and their TRANSFAC counterparts. There were some similarities between the discovered motifs and the three known PREs from TRANSFAC (p<5×10^−5^). We noted, however, novel and unique features that were common to all three discovered motifs. There was a clear preference for a G in positions 1 and 10, an A in positions 3 and 5, a C in positions 4 and 13, and a T in positions 9 and 11. Based on the discovered sequences from our genome-wide PR binding data, we propose the following revised consensus PRE: G•ACA•••TGT•C (Figure 2D). This 13 bp revised motif adheres to the classical consensus frame comprised of two palindromic repeats separated by interchangeable 6th, 7th, and 8th spacer nucleotides. Here, we found that adenines and thymidines at the 2nd and 12th bp positions are highly variable and possibly dispensable.

An analysis of the top five discovered motifs with the highest similarity to a known motif in the TRANSFAC or JASPAR databases identified UF1-H3β, SP-1, AR, Krox, and ZFP281 binding motifs (Figure S1). The UF1-H3β motif is a sequence in the FOXA2 promoter (*Mus musculus*) that binds a ubiquitous DNA binding protein [50]; the Krox element has been found in the human tumor necrosis factor promoter [51]; and ZFP281 binds to the G-rich box in the enhancer region of ornithine decarboxylase, an essential enzyme for cell growth [52].

### Characterization of genes with TSSs within 50 kb of a PR-binding site

We performed KEGG molecular pathway enrichment analysis on three sets of genes. The first two sets, one for breast cancer and one for leiomyoma, contained genes whose TSS was located within 50 kb of a PR-binding site in each cell type. The third set focused on genes with TSSs within 50 kb of any of the PR-binding sites that were common to both cell types. Our goal was to determine the functional associations of genes within 50 kb of a PR-binding site.

Of the genes with PR-binding sites common to both cell types, several KEGG pathways were overrepresented, including those of focal adhesion, regulation of actin cytoskeleton, neurotrophin signaling, ErbB signaling, various cancer pathways, and insulin signaling (Table 3).

**Table 3:** KEGG pathways enriched in genes with TSSs located within 50 kb of a PR-binding site.

KEGG ID	Name	p-value	FDR
	**Pathways enriched in both cell types**		
4510	Focal adhesion	2.1e-04	5.9e-03
4810	Regulation of actin cytoskeleton	7.1e-05	4.6e-03
4722	Neurotrophin signaling pathway	8.8e-04	1.2e-02
5215	Prostate cancer	1.7e-04	6.1e-03
5220	Chronic myeloid leukemia	1.5e-03	1.6e-02
4012	ErbB signaling pathway	3.3e-04	7.4e-03
4670	Leukocyte transendothelial migration	1.3e-03	1.5e-02
5200	Pathways in cancer	8.3e-04	1.0e-02
5211	Renal cell carcinoma	9.0e-04	1.2e-02
4910	Insulin signaling pathway	1.0e-03	1.0e-02
5221	Acute myeloid leukemia	5.2e-03	4.4e-02
5222	Small cell lung cancer	1.6e-03	1.6e-02
5212	Pancreatic cancer	3.4e-03	2.3e-02
4520	Adherens junction	6.9e-03	4.0e-02
	**Pathways enriched in T47D breast cancer cells only**		
4144	Endocytosis	3.2e-06	4.4e-04
4120	Ubiquitin-mediated proteolysis	4.5e-06	4.4e-04
4150	mTOR signaling pathway	1.8e-04	5.8e-03
0603	Glycosphingolipid biosynthesis - globo series	5.2e-04	1.0e-02
4360	Axon guidance	7.0e-04	1.2e-02
4530	Tight junction	1.1e-03	1.3e-02
4310	Wnt signaling pathway	3.4e-03	3.4e-02
4210	Apoptosis	3.6e-03	3.4e-02
0512	O-glycan biosynthesis	4.0e-03	3.6e-02
1100	Metabolic pathways	5.5e-03	4.5e-02
	**Pathways enriched in leiomyoma cells only**		
4512	ECM-receptor interaction	1.3e-07	1.2e-05
4666	Fc gamma R-mediated phagocytosis	2.5e-04	7.3e-03
4630	Jak-STAT signaling pathway	4.2e-04	7.6e-03
4960	Aldosterone-regulated sodium reabsorption	7.2e-04	1.0e-02
4070	Phosphatidylinositol signaling system	9.5e-04	1.0e-02
5214	Glioma	1.0e-03	1.0e-02
0562	Inositol phosphate metabolism	1.0e-03	1.0e-02
5223	Non-small cell lung cancer	1.0e-03	1.0e-02
5410	Hypertrophic cardiomyopathy (HCM)	1.6e-03	1.4e-02
5213	Endometrial cancer	2.1e-03	1.7e-02
4662	B-cell receptor signaling pathway	2.1e-03	1.7e-02
5218	Melanoma	2.9e-03	2.1e-02
4010	MAPK signaling pathway	2.9e-03	2.1e-02
4062	Chemokine signaling pathway	3.7e-03	2.5e-02
5414	Dilated cardiomyopathy	4.3e-03	2.8e-02
5412	Arrhythmogenic right ventricular cardiomyopathy (ARVC)	6.0e-03	3.8e-02
4660	T-cell receptor signaling pathway	6.4e-03	3.9e-02

Although PR may affect these functions in both cell types, we also identified several KEGG pathways that were unique to each cell type. In T47D breast cancer cells, pathways involving endocytosis, ubiquitin mediated proteolysis, mTOR signaling, Wnt signaling, and apoptosis were specifically overrepresented (Table 3). On the other hand, pathways involving extracellular matrix (ECM)-receptor interaction, Fc gamma R-mediated phagocytosis, Jak-STAT signaling, glioma, and hypertrophic cardiomyopathy were specifically overrepresented in leiomyoma cells (Table 3). These data suggest that distinct pathways can be activated by an antiprogestin and PR in a cell-specific manner.

### Genes regulated by RU486 in breast cancer and leiomyoma cells

**Figure 3:**
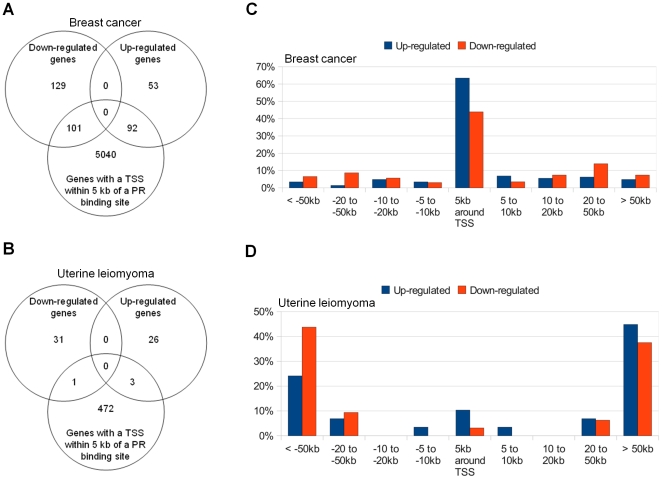
Correlation between RU486-induced PR-binding and RU486-mediated gene expression. Venn diagrams summarize the number of RU486-mediated genes with identified PR-binding sites within 5 kb from their TSSs in breast cancer cells (A) and leiomyoma cells (B). Distribution of PR-binding sites relative to TSSs of differentially expressed genes in T47D breast cancer cells (C) and uterine leiomyoma cells (D). T47D and leiomyoma cells were treated with vehicle (ethanol) or RU486 (10^−6^ M) for 6 hours and expression profiling was performed using Human HT-12 v4 Expression BeadChip arrays from Illumina. The percentage of all differentially regulated genes with PR-binding sites in each region is shown in the vertical axis. The distance relative to the TSS is shown in the horizontal axis.

To correlate RU486-induced PR binding with RU486-mediated gene regulation, we performed microarray gene profiling assays of T47D breast cancer cells and primary leiomyoma cells treated with RU486 or vehicle for 6 hours. We identified 230 downregulated and 145 upregulated genes in response to RU486 treatment in breast cancer cells (Figure 3A), whereas RU486 treatment downregulated 32 and upregulated 29 genes in leiomyoma cells (Figure 3B). Complete lists of these genes are presented in Tables S4 (breast cancer cells) and S5 (leiomyoma cells). RU486 regulated seven genes in both cell types: prothymosin alpha (PTMA), SWI/SNF-related matrix-associated actin-dependent regulator of chromatin subfamily a member 1 (SMARCA1), capping protein (actin filament) muscle Z-line alpha 1 (CAPZA1), perilipin 2 (PLIN2), poly(A)-binding protein cytoplasmic 1 (PABPC1), annexin A2 pseudogene 1 (ANXA2P1), and tryptophan-rich basic protein (WRB). RU486 upregulated PLIN2 and downregulated the rest of these genes in both cell types. Except for ANXA2P1 and WRB, which have unknown functions, all of these genes have been reported to play important roles in cellular function [53], [54], [55]. For example, PTMA, a downstream target gene of estrogen, encodes an oncoprotein associated with cell proliferation and chromatin remodeling [56]. SMARCA1, a chromatin remodeling gene, has been reported to stimulate steroidogenic acute regulatory (StAR) protein expression through association with the StAR promoter, and SMARCA1 knockdown causes significant growth inhibition and induces apoptosis of cancer cells [57], [58].

The distribution of PR-binding sites relative to the nearest TSS of RU486-regulated genes is shown in Figure 3C for the T47D breast cancer cells and Figure 3D for leiomyoma cells. A striking difference was observed between the two cell types. In breast cancer cells, the majority of the differentially regulated genes (51.47%) contained a PR-binding site within 5 kb up- or downstream of their TSSs. In contrast, the majority of the RU486-regulated genes (75.41%) in leiomyoma cells had a PR-binding site more than 50 kb away from the TSSs. To further compare the differences in proximal regulatory element usage between the two cell types, we examined the genomic regions located 5 kb up- and downstream of the TSSs of differentially expressed genes. In breast cancer cells, 43.9% of downregulated genes and 63.4% of upregulated genes contained PR-binding sites within 5 kb from their TSSs (Figure 3C). This finding indicates the significant enrichment of PR-binding sites in genomic regions proximal to the TSSs of RU486-regulated genes in breast cancer cells. In contrast, only 10.3% of upregulated and 3.1% of downregulated genes contained PR-binding sites within 5 kb of their TSSs in leiomyoma cells (Figure 3D). Taken together, these findings indicate the presence of distinct mechanisms that regulate RU486/PR action in breast cancer cells *vs.* leiomyoma cells. RU486/PR-mediated regulation in breast cancer cells favors proximal regulatory elements, whereas distal enhancer elements are used more often in leiomyoma cells.

### RU486 regulates PLIN2 expression in a dose- and time-dependent manner

We selected the PLIN2 gene that encodes adipophilin to validate its regulation by RU486. PLIN2 contains PR-binding sites that overlap with its TSS, and we showed that it was upregulated by RU486 in both cell types. PLIN2 plays key roles in lipid droplet formation and milk formation and secretion [59], [60]. High PLIN2 expression is associated with better cancer-free survival in clear cell renal carcinoma [61]. PLIN2 is also involved in the regulation of fibrotic genes, including the suppression of collagen I and matrix metalloproteinase-2 mRNA levels and stimulation of matrix metalloproteinase-1 mRNA levels [55].

**Figure 4:**
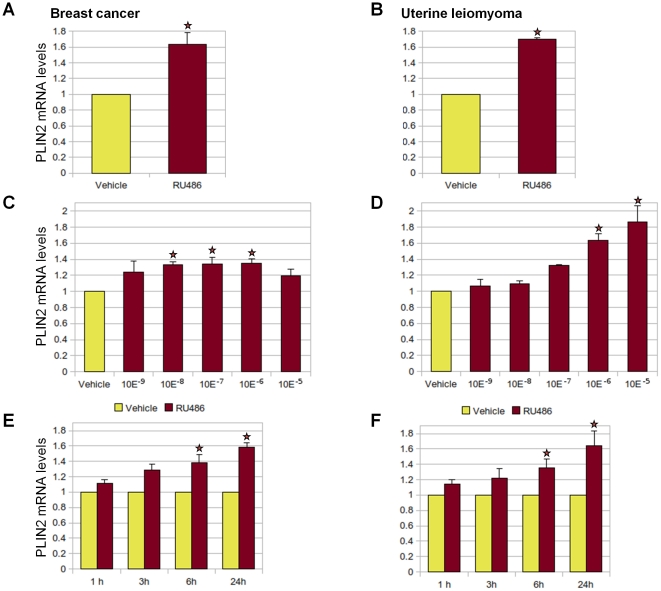
Effect of RU486 treatment on the expression of PLIN2. Expression of PLIN2 by real-time PCR in breast cancer T47D (A) and primary uterine leiomyoma cells (B). Serum-starved T47D or leiomyoma cells were stimulated with variable concentrations of RU486 (ranging from 10^−9^ to 10^−5^ M) or vehicle for 6 hours (C and D), or RU486 (10^−6^ M) or vehicle for 1, 3, 6, or 24 hours (E and F). PLIN2 mRNA levels were normalized to GAPDH expression. Results are reported as fold change compared with cells treated with vehicle only and represent the mean ± SEM of three independent experiments. Reference: star symbol, p<0.05 compared with vehicle treatment.

As shown in Figure 4A and 4B, we used real-time PCR to verify the changes in PLIN2 mRNA that we observed in our microarray analysis. RU486 (10^−6^ M) treatment for 6 hours increased PLIN2 expression in T47D breast cancer cells and leiomyoma cells. To analyze the dose response of PLIN2 expression to RU486 treatment in both cell types, various concentrations (10^−9^ to 10^−5^ M) of RU486 or vehicle (ethanol) were added to the medium for 6 hours. The greatest induction in PLIN2 expression was observed at 10^−7^ M RU486 in T47D breast cancer cells and 10^−5^ M RU486 in leiomyoma cells (Figure 4C and 4D). To explore the temporal response of PLIN2 expression to RU486 stimulation, both cell types were treated with 10^−6^ M of RU486 for 1, 3, 6, or 24 hours. RU486 stimulated PLIN2 mRNA levels as early as 6 hours, and increased throughout the 24 hour treatment time point in both cell types (Figure 4E and 4F).

## Discussion

Progesterone is extraordinarily important to normal reproductive function, and acts as a master regulator to control implantation, maintenance of pregnancy, and breast development, as well as inhibit endometrial carcinogenesis. Progesterone has also been implicated in the promotion of growth of breast cancer and uterine leiomyoma. The anti-progestin RU486 inhibits both breast cancer and uterine leiomyoma growth and thus represents a potential therapeutic for these reproductive diseases.

ChIP-seq has been used successfully to identify genome-wide ER- and GR-binding sites [30], [32], [33], yet no such literature is available for PR despite the important and complex effects of progesterone on the female reproductive tract. To unravel the target gene network of a particular TF, in this case PR, the identification of interaction sites, cis-regulatory elements, and target genes is essential. Therefore, we applied ChIP-seq to identify PR-interaction sites in T47D breast cancer cells and primary uterine leiomyoma cells treated with RU486. Using a relatively stringent analytic approach, we found 31,457 PR-binding sites in breast cancer cells and 7,034 in leiomyoma cells, with 1,035 PR-binding sites that overlapped between the two cell types. Microarray gene profiling analyses found seven genes commonly regulated by RU486 in both cell types. Based on the function of these genes and the same direction of the regulation, these findings provide insight into the mechanisms underlying the observed clinical effects of RU486 treatment in these diseases.

Using cell-free assays or transfection of gene reporter constructs, the consensus sequence for binding of GR, PR, AR, and MR (mineralocorticoid receptor) has been predicted to be an inverted repeat with a three-nucleotide spacer region: AGAACAnnnTGTTCT, where the underlined nucleotides are most important for receptor binding [62]. Using insertion and substitution of the PRE core sequence in the mouse mammary tumor virus promoter in in vitro PR binding assays, Lieberman et al proposed an optimal PR binding motif as −7RGNACANRNTGTNCY+7 [63]. Here, we report the first in situ determination of a consensus PRE based on ChIP-seq data from 1,035 PR-binding sites in both breast cancer and leiomyoma cells: G•ACA•••TGT•C, which is consistent with the two previously reported motifs. The finding that the independently determined consensus sequence from the ChIP-seq data closely matched the previously defined optimal binding sites for PR supports the biological relevance of our ChIP-seq results.

In addition to the classical complete PRE, PR has also been reported to regulate gene transcription through interaction with half PRE sites [39]. Supporting this notion, we found that genome-wide PR targets contained strikingly higher numbers of half PREs compared with full length PRE motifs. It has been demonstrated that the magnitude of estrogen-dependent recruitment of ER to binding sites containing canonical full EREs is stronger than recruitment to sites containing half-EREs [33]. It will be interesting to clarify whether PR has the same characteristics. The mechanisms by which endogenous PR prefers to bind half PRE are unknown. Thus far, we cannot exclude the possibility that the abundance of half PRE sites in native chromatin is much higher than that of full PRE sites.

Interestingly, de novo motif analysis identified UF1-H3β as one of the most highly enriched motifs among genome-wide PR-binding sites. Although it is not clear what TF binds to this sequence, the UF1-H3β motif is also overrepresented in the promoters of direct targets of FOXA2. It has been reported in M. musculus that proximal UF1-H3β and SP-1 binding sites are involved in repressing FOXA1 promoter activity [64]. The functional importance of the enrichment of this motif in PR-binding sites is unclear.

In line with observations that PR physically and/or functionally interacts with other TFs, we revealed that, among others, the FOX family member motifs are specifically enriched at PR targets in breast cancer cells, whereas HSF, TEF-1, and C/EBP motifs are overrepresented in leiomyoma cells, suggesting that through interaction with those TFs, PR may produce cell-specific effects. Others have reported that FOXA1 is enriched in binding sites of ER and GR, substantiating the notion that this TF may be a key factor that coordinates with nuclear receptors of steroid hormones to regulate their downstream gene targets [22], [45], [46]. Among the top 20 TF binding motifs enriched at genome-wide PR targets, we found that AP2 and SP1 sites were the most frequent motifs in breast cancer cells, whereas AP1 was the most frequent motif in leiomyoma cells. Given that these motifs bind common TFs that interact with PR in the absence of a PRE, the mechanism underlying the cell-specific preference of their usage is unknown.

In breast cancer cells and leiomyoma cells, we found that PR interaction sites significantly overlapped. Many of the genes annotated with these PR-binding sites are critical regulators of cell migration, metabolic processes, and biosynthetic processes (Yin P and Bulun SE, unpublished data), and affect pathways including ErbB and insulin signaling, as well as pathways in cancer. Through KEGG pathway enrichment analysis, we also identified several pathways uniquely enriched in each cell type, suggesting that PR may exert specific functional effects on each disease. In breast cancer, which is characterized by rapid growth, invasion, and metastasis, PR was associated with pathways involving mTOR and Wnt signaling and apoptosis. In contrast, leiomyoma cells are characterized by slow growth and overproduction of ECM proteins, and PR may specifically affect the interaction between cell surface receptors and ECM elements in this tissue.

Consistent with earlier studies [7], [30], [42], [43], a small fraction of PR-interaction sites were located in promoter regions, whereas the majority of sites was found at large distances from annotated genes or within introns. These distal sites most likely act as enhancers and interact with receptive promoters through looping to regulate gene expression, as has been described for PR target genes such as E2F1 and KLF11, and ERα target genes such as TFF1, GREB1, and bcl-2 [7], [22], [65], [66], [67].

Compared with leiomyoma cells, RU486 treatment of T47D breast cancer cells induced a notably higher number of genome-wide PR interaction sites and differentially regulated mRNA species. These observations may be partially due to the significantly higher PR protein level in T47D breast cancer cells than in leiomyoma cells (Yin P and Bulun SE, unpublished data). Moreover, the amount of chromatin material that can potentially interact with PR is higher in T47D cells, which have 66 chromosomes rather than the usually normal karyotype of leiomyoma cells [68].

Traditionally, PR has been characterized as a TF that binds to specific DNA sequences in the regulatory regions of genes. Our data provides the first evidence that there is a cell-type specific preference in the usage of proximal regulatory regions vs. distal enhancer regions in the regulation of gene transcription by PR. In T47D breast cancer cells, 51.47% of RU486-regulated genes contained a PR-binding site within 5 kb up- or downstream from their TSSs, whereas PR binding proximal to a TSS was associated with only 6.56% of RU486-regulated genes in leiomyoma cells. One explanation for this cell-type specific preference is that the receptors interact or cooperate with other TFs and that this cooperation may be in part determined by the availability of these TFs in the specific cell type. In addition, the local chromatin landscape and histone modifications in each cell type are likely to play decisive roles in gene regulation, as has been described for GR [69].

It has been reported that the majority of estrogen-activated genes are associated with one or more ERα-binding sites, indicating that ERα complexes bound to multiple interaction sites probably cooperate to regulate expression of diverse target genes such as TCF4 and MYC [70], [71]. PR may use the same mechanisms to mediate its target gene expression. Nevertheless, the requirement of multiple PR-binding sites per RU486-regulated gene alone cannot account for the extreme discordance between the number of RU486-regulated genes (375) and PR-binding sites (31,457) in T47D cells, and the number of RU486-regulated genes (61) and PR-binding sites (7,034) in leiomyoma cells. This is exemplified in Figure 1B showing PR-binding activity in close proximity to ten genes, only four of whose mRNA levels are regulated by RU486 in breast cancer cells. It appears that the dispersed and often distal localization of PR-binding sites complicates the assignment of PR-interaction sites to RU486-responsive genes.

More importantly, mRNA levels do not necessarily reflect gene activity because transcripts are subject to degradation and regulation, and not all PR-binding sites are likely to be active under all conditions. In this case, genome-wide profiling of RNA polymerase II occupancy might provide a much more direct readout and could yield insights beyond what is typically obtained by mRNA expression profiling [30]. Also, the inherent differences between analyses based on PR occupancy and on mRNA levels cannot be ignored.

In conclusion, our study provides novel and important insights into the regulation of the PR target gene network and serves as a resource for the further elucidation of PR-regulated transcription in T47D and leiomyoma cells. Our findings may directly inform the development of common and disease-specific treatment strategies for human breast cancer and uterine leiomyoma.

## Materials and Methods

### Ethics Statement

To obtain human tissues, we followed the protocol approved by the Institutional Review Board for Human Research of Northwestern University. Written informed consent was received from all subjects.

### Tissue collection and cell culture

Human uterine leiomyoma tissues were obtained at surgery from 20 premenopausal women (mean age 40 years, range 33–48). The subjects had not received any hormonal treatment during the six months prior to surgery. The size of the tumors varied between 3.5 to 10 cm and their location was predominantly intramural. Primary leiomyoma cells were cultured as previously described [72]. Cells used in these experiments were passaged one or two times. T47D breast cancer cells were kindly provided by Dr. Frasor J (University of Illinois at Chicago) and were cultured as previously described [73].

### ChIP-seq

T47D or leiomyoma cells treated with RU486 for 1 hour were fixed with 1% formaldehyde for 15 min and quenched with 0.125 M glycine. Chromatin was isolated by adding lysis buffer, followed by disruption with a Dounce homogenizer. Lysates were sonicated and the DNA sheared to an average length of 300–500 bp. Genomic DNA was prepared by treating aliquots of chromatin with RNase, proteinase K and heat for de-crosslinking, followed by ethanol precipitation. Pellets were resuspended and the resulting DNA was quantified on a NanoDrop spectrophotometer. Extrapolation to the original chromatin volume allowed quantitation of the total chromatin yield.

An aliquot of chromatin (30 µg) was precleared with protein G agarose beads (Invitrogen). Genomic DNA regions of interest were isolated using 4 µg antibody against PR (sc-7208, Santa Cruz Biotechnology). Complexes were washed, eluted from the beads with SDS buffer, and subjected to RNase and proteinase K treatment. Crosslinks were reversed by incubation overnight at 65°C, and ChIP DNA was purified by phenol-chloroform extraction and ethanol precipitation.

We followed the procedures for preparing the Illumina genomic DNA library for sequencing, and the resulting DNA from the leiomyoma and T47D cells was sequenced separately on the Illumina Genome Analyzer 2 (Illumina, Inc.). The identified 35-nt sequence reads were mapped to the NCBI GRCh37 assembly of the human genome using the Solexa GAPipeline and the ELAND standalone program [http://www.illumina.com/software/genome_analyzer_software.ilmn#casava]. Only reads that mapped uniquely, had no more than two mismatches, and passed quality control filtering were considered. The enriched binding sites were identified with the Model-based Analysis of ChIP-seq Algorithm (MACS) [74]. MACS models the distribution of tags across the genome with a Poisson distribution obtained from the control sample. Because the experiment does not have a control dataset, the background distribution of tags was modeled using the reads in the ChIP-seq samples. The tag size was set at 36 nt and the bandwidth was set to 130 nt. A conservative p-value of 10^−11^ was used to call peaks. In order to keep the false discovery rate (FDR) low, and because of the absence of a control sample, no global lambda was used.

### Mapping genes adjacent to PR-binding sites

To determine the association of PR-binding sites with genes, we computed the distance of each binding site to the closest TSS. Genomic locations were obtained for all gene transcripts in the UCSC RefSeq track of the latest Human Genome Assembly: hg19/GRCh37 [41]. Distance between a binding site and a transcript's TSS was computed using the bindSDb database, which takes into consideration the strand orientation [75]. The minimum distance between a PR binding site and a TSS was used to determine association between the gene and the binding site.

### Validation of the *in vivo* PR-binding sites by ChIP-real time PCR

Standard ChIP assays followed by real-time PCR were used to validate ten PR-binding sites identified by ChIP-seq. T47D or leiomyoma cells were treated with RU486 (10^−6^ M) or vehicle (ethanol) for 1 hour. DNA after ChIP of PR and IgG (ab46540, Abcam Inc.) as well as input DNA were analyzed by real time PCR using SYBR Green Master Mix (Applied Biosystems). Primers were generated corresponding to the regions within each ChIP-seq derived genomic fragment. Their sequences are available upon request. The RU486-mediated fold enrichment of PR-binding regions relative to IgG was compared with its vehicle control.

### Motif search

All PR-binding sites were resized to a length of 1,000 bp (500 bp to the left and right of the peak) and their nucleotide sequences were obtained from the UCSC hg19 genome assembly. These sequences were analyzed to identify common motifs using two different approaches: (1) a search for known motifs using position weight matrices (PWMs) and (2) *de novo* discovery of motifs.

#### Motif search using PWMs

All PR-binding sites were scanned with the MATCH software using the PWMs from TRANSFAC (ver. 2010.1) [44], [76]. The set of thresholds labeled as “Minimize False Positive” was used to identify putative TF binding sites. To evaluate the significance of motif enrichment, we compared the enrichment of that motif against a set of background sequences. For each PR-binding site, a background site of the same length was created from its vicinity (within a random distance of 1 to 10 kb up- or downstream and not overlapping with any other true PR-binding site). The nucleotide sequences for each of these background sites were obtained. Then, as it was performed for the PR-binding sites, each PWM from TRANSFAC was scanned in the background sites using the MATCH algorithm and the “Minimize False Positive” set of thresholds. The choice of using true DNA sequences for our background set, as opposed to synthetic ones, was based on our goal to have a better model of binding affinity for PWMs.

For each PWM, the proportion of nucleotides from the true binding sites that contained a reported hit of that PWM was obtained and compared with the proportion of nucleotides in background sites that also contained a hit. For a large sample size, the central limit theorem indicates that the distribution of the sample proportion is approximately normally distributed. Therefore, a *z*-value was computed for a PWM according to formula (1):
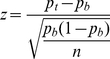
(1)where:

*p_t_* is the proportion of nucleotides from true PR-binding sites where the PWM scored a hit.
*p_b_* is the same proportion as above but for nucleotides in the background sites.
*n* is the total number of nucleotides (number of binding sites×length of binding sites).

Based on the *z*-value for each PWM, the p-value was computed and adjusted for multiple hypotheses testing using the Bonferroni correction.

#### Progesterone receptor PWM (motif)

TRANSFAC [44] normally has one or more PWMs to evaluate the binding affinity of one TF. In the case of PR, there are three different PWMs: PR_02, progesterone receptor; PR_01, obtained from high affinity binding sites; and PR_Q2, the half-site PRE. In our motif analysis, a binding site was considered to have a PR motif if MATCH reported at least one hit for any one of the three PWMs.

#### De novo discovery of motifs

In addition to the enrichment analysis with PWMs, a motif discovery analysis using MEME was conducted [48]. We focused only on the PR-binding sites that were common to both cell types. Numerous scans on these sites were run, changing the width of the target motif from 8 to 23, and looking not only at the target sequence, but also at its reverse complement. For each run, the top 20 motifs were selected (based on their p-values). The output of all the runs was then consolidated using the motif comparison tool Tomtom [77]. Tomtom takes a query motif, compares it to a target motif, and provides a statistical measure (p-value) of how similar they are compared to a null model. When the query is compared to multiple targets, the p-value is corrected for multiple hypotheses testing, thereby yielding an E-value. A third metric, called a q-value, measures the minimum FDR necessary to report the match between the motifs. In our analysis, when multiple discovered motifs had high similarity to a known TRANSFAC or JASPAR [49] motif, the one with the lowest E-value was kept. The q-value was used to rank all matches.

### Gene functional annotation analysis

Gene set enrichments of KEGG pathways were determined using hypergeometric tests. Two sets of genes were analyzed for KEGG enrichment: genes whose TSS was located within 50 kb of PR-binding sites in T47D cells, genes whose TSS was located within 50 kb of PR-binding sites in leiomyoma cells. A hypergeometric test was performed on each set to determine overrepresentation in KEGG pathways. The gene universe for the KEGG pathways was 5,501 genes uniquely identified by Entrez ID and with the condition of having at least one KEGG pathway associated with them. The enriched KEGG pathways in each set with FDR less than 0.05 were reported. The analysis was implemented with the GOstats package in Bioconductor.

When listing in Table 3 the pathways that were enriched in both hypergeometric tests, the largest p-value and FDR values, for each pathway, were reported.

### Microarrays and data analysis

Total RNA was isolated from T47D or leiomyoma cells treated with vehicle (ethanol) or RU486 (10^−6^ M) for 6 hours. Expression profiling was performed using HumanHT-12 v4 Expression BeadChip arrays from Illumina (Illumina, Inc.) following the protocol described previously [78]. For each cell type, six microarrays were processed: three vehicle and three treated samples. After quality control, three of the samples were discarded from further analysis (one vehicle and one treatment for T47D cells, and one treatment for leiomyoma cells). The raw expression data were normalized using the Robust Spline Normalization (RSN) algorithm implemented in the lumi package in Bioconductor [79]. Only the probes with a present call were considered for the analysis. The differentially expressed probes were determined by the fold change (FC) of expression level in RU486 treated *vs.* control. The FC threshold was set to ±1.40 for T47D cells and ±1.30 for leiomyoma cells. Finally, probes were mapped to genes using the nucleotide universal Identifier (nuID) annotation. The microarray data is MIAME compliant and is available at the Gene Expression Omnibus Web site (http://www.ncbi.nlm.nih.gov/geo) under accession number GSE30871.

### Real-time quantitative PCR

Total RNA from T47D and leiomyoma cells was extracted using Tri-reagent (Sigma-Aldrich). Complementary DNA was prepared with Superscript III first-Strand Synthesis System (Invitrogen). PLIN2 and glyceraldehyde-3-phosphate dehydrogenase (GAPDH) mRNA were amplified by real-time PCR using SYBR Green Master Mix. The primer sequences used for PCR were: GAPDH: forward: 5′-GAAGGTGAAGGTCGGAGTC-3′; reverse: 5′-GAAGATGGTGATGGGATTTC-3′; PLIN2: forward: 5′-AGTATCCCTACCTGAAGTCTGTG-3′; reverse: 5′-CCCCTTACAGGCATAGGTATTG-3′. All gene expressions were normalized to GAPDH.

### Statistical analysis

The Fisher's exact test was used to test the enrichment of PR-binding sites in proximity of genomic regions of TSSs for the differentially expressed genes. Differences between groups were analyzed by Student's *t* test or one-way ANOVA analysis followed by Fisher's protected least significance difference test. Significance was accepted at p<0.05.

## Supporting Information

Figure S1 ***De novo*** **search of** ***cis*** **-regulatory motifs enriched within PR-binding sites.** MEME analysis identified putative motifs for other TFs in ChIP-seq data from the 1,035 PR-binding sites common to both T47D breast cancer cells and leiomyoma cells. The top five motifs (lower panels) with the highest similarity to a known motif in TRANSFAC or JASPAR (upper panels) are shown in A, B, C, D, and E. Vertical axes (Bits) indicate the information content of the base frequency at that position. The horizontal axes refer to consensus site position.
(TIF)

Table S1 **Genomic distributions of PR-binding sites with respect to their closest TSS in T47D breast cancer cells and leiomyoma cells.**
(DOC)

Table S2 **Top 20 enriched TF binding motifs in PR-binding sites in T47D breast cancer cells but not in leiomyoma cells.**
(DOC)

Table S3 **Top 20 enriched TF binding motifs in PR-binding sites in leiomyoma cells but not in breast cancer cells.**
(DOC)

Table S4 **Genes regulated by RU486 in T47D breast cancer cells.**
(XLS)

Table S5 **Genes regulated by RU486 in leiomyoma cells.**
(XLS)
