# Supplementary figures and images for: Genome-Wide Progesterone Receptor Binding: Cell Type-Specific and Shared Mechanisms in T47D Breast Cancer Cells and Primary Leiomyoma Cells
Source: PLoS One. 2012 Jan 17;7(1):e29021. doi: 10.1371/journal.pone.0029021 (PMC3260146; doi:10.1371/journal.pone.0029021)

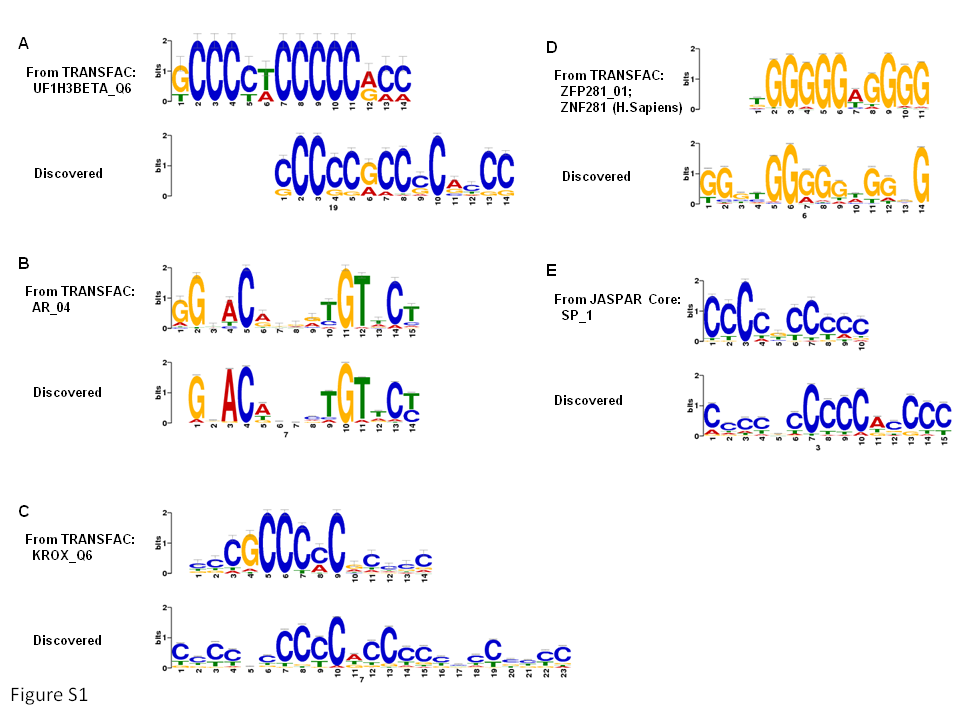

Supplement: Figure S1 — De novo search of cis -regulatory motifs enriched within PR-binding sites. MEME analysis identified putative motifs for other TFs in ChIP-seq data from the 1,035 PR-binding sites common to both T47D breast cancer cells and leiomyoma cells. The top five motifs (lower panels) with the highest similarity to a known motif in TRANSFAC or JASPAR (upper panels) are shown in A, B, C, D, and E. Vertical axes (Bits) indicate the information content of the base frequency at that position. The horizontal axes refer to consensus site position. (TIF) [file pone.0029021.s001.tif]
