# Supplementary material for: Genome-Wide Progesterone Receptor Binding: Cell Type-Specific and Shared Mechanisms in T47D Breast Cancer Cells and Primary Leiomyoma Cells
Source: PLoS One. 2012 Jan 17;7(1):e29021. doi: 10.1371/journal.pone.0029021 (PMC3260146; doi:10.1371/journal.pone.0029021)
Supplement: Table S1 — Genomic distributions of PR-binding sites with respect to their closest TSS in T47D breast cancer cells and leiomyoma cells. (DOC) [file pone.0029021.s002.doc]

# Supplemental Table S1

Genomic distributions of PR-binding sites with respect to their closest TSS in T47D breast cancer cells and leiomyoma cells.

|  | | **T47D** | | **Leiomyoma** | |
| --- | --- | --- | --- | --- | --- |
| < -50 kb | | 4560 | 14.50% | 1280 | 18.20% |
| -20 to -50 kb | | 2119 | 6.74% | 455 | 6.47% |
| -10 to -20 kb | | 1302 | 4.14% | 228 | 3.24% |
| -5 to -10 kb | | 833 | 2.65% | 180 | 2.56% |
| 5 kb around TSS | 5 kb upstream of TSS | 3418 | 10.87% | 244 | 3.47% |
|  | 5 kb downsteam of TSS | 1756 | 5.58% | 198 | 2.81% |
| 5 to 10 kb | | 1170 | 3.72% | 227 | 3.23% |
| 10 to 20 kb | | 2073 | 6.59% | 381 | 5.42% |
| 20 to 50 kb | | 3894 | 12.38% | 872 | 12.40% |
| > 50 kb | | 10332 | 32.84% | 2969 | 42.21% |

For genes with multiple TSSs, the TSS that was closer in absolute distance to the binding site was chosen. If more than one TSS had the same distance to the binding site, the TSS with the smaller RefSeq ID was chosen.
