# Supplementary material for: Genome-Wide Progesterone Receptor Binding: Cell Type-Specific and Shared Mechanisms in T47D Breast Cancer Cells and Primary Leiomyoma Cells
Source: PLoS One. 2012 Jan 17;7(1):e29021. doi: 10.1371/journal.pone.0029021 (PMC3260146; doi:10.1371/journal.pone.0029021)
Supplement: Table S2 — Top 20 enriched TF binding motifs in PR-binding sites in T47D breast cancer cells but not in leiomyoma cells. (DOC) [file pone.0029021.s003.doc]

# Supplemental Table S2

Top 20 enriched TF binding motifs in PR-binding sites in T47D breast cancer cells but not in leiomyoma cells (based on a subset of the 100 most highly ranked TRANSFAC matrices for each cell type).

| **Factor name** | **Description** | **Motifs (TRANSFAC**  **matrix ID)** | **Z-value** | **p-value** |
| --- | --- | --- | --- | --- |
| AP-2 α,β,γ | Activator protein 2 α, β, and γ | V$AP2ALPHA_03 | 81.298 | < 1.0e-323 |
|  |  | V$AP2ALPHA_02 | 58.466 | < 1.0e-323 |
|  |  | V$AP2GAMMA_01 | 53.483 | < 1.0e-323 |
|  |  | V$AP2_Q6 | 50.987 | < 1.0e-323 |
|  |  | V$AP2_Q6 | 50.987 | < 1.0e-323 |
|  |  | V$AP2_Q6_01 | 41.854 | < 1.0e-323 |
|  |  | V$AP2_Q3 | 40.629 | < 1.0e-323 |
| SP 1,2,3 | Stimulating proteins 1, 2, and 3; SP1; SP2; SP3 | V$SP1_Q6 | 82.245 | < 1.0e-323 |
|  |  | V$SP1_Q4_01 | 80.650 | < 1.0e-323 |
|  |  | V$SP1_Q6_01 | 79.594 | < 1.0e-323 |
|  |  | V$SP1_Q2_01 | 62.416 | < 1.0e-323 |
|  |  | V$SP1_01 | 53.875 | < 1.0e-323 |
|  |  | V$SP1_02 | 53.385 | < 1.0e-323 |
| ETF | EGFR-specific transcription factor | V$ETF_Q6 | 81.369 | < 1.0e-323 |
| Ben | Anti-general transcription factor 3; GTF3 | V$BEN_01 | 67.708 | < 1.0e-323 |
| FREAC 2,4 | Forkhead related activator-2; FOXF2, FREAC2, | V$FREAC4_01 | 59.314 | < 1.0e-323 |
|  | and FREAC4 | V$FREAC2_01 | 58.989 | < 1.0e-323 |
| ChCh | Churchill protein | V$CHCH_01 | 55.380 | < 1.0e-323 |
| Zinc | Zinc finger protein 333; ZNF333 | V$ZNF333_01 | -55.338 | < 1.0e-323 |
| GKLF | Epithelial zinc-finger protein; EZF; Krueppel-like factor 4 | V$GKLF_02 | 55.043 | < 1.0e-323 |
| HNF3A | FOXA1; Hepatocyte nuclear factor 3 alpha; HNF3A | V$HNF3A_01 | 53.536 | < 1.0e-323 |
| Elk-1 | Elk-1; p62TCF | V$ELK1_02 | 53.049 | < 1.0e-323 |
| E2F-1,2,3,4 | E2F transcription factor 1, 2, 3, and 4 | V$E2F_Q2 | 52.318 | < 1.0e-323 |
| Otx3 | Diencephalon/mesencephalon homeobox 1; Dmbx1 | V$OTX3_01 | -50.794 | < 1.0e-323 |
| Sp4 | Transcription factor Sp4; SPR-1 | V$SP4_Q5 | 49.438 | < 1.0e-323 |
| FOXO1A | Forkhead box O1A (rhabdomyosarcoma); FOXO1 | V$FOXO1_02 | 48.892 | < 1.0e-323 |
| Egr-1,2,4 | Early growth response proteins 1, 2, and 4; EGR2; EGR4 | V$KROX_Q6 | 44.484 | < 1.0e-323 |
| GABP-α | GA binding protein alpha subunit; E4TF1-60 | V$GABPALPHA_Q4 | 43.250 | < 1.0e-323 |
| Barx-2 | BarH-like homeobox 2; BARX2 | V$BARX2_01 | -42.843 | < 1.0e-323 |
| Ets-1 | C-Ets-1 proto-oncogene; c-Ets-1; p54 | V$CETS1P54_03 | 41.179 | < 1.0e-323 |
| LHX4 | LIM/homeobox protein Lhx4 | V$LHX4_01 | -40.144 | < 1.0e-323 |
| En-1 | Engrailed 1 | V$EN1_02 | -39.303 | < 1.0e-323 |
