# Supplementary material for: Genome-Wide Progesterone Receptor Binding: Cell Type-Specific and Shared Mechanisms in T47D Breast Cancer Cells and Primary Leiomyoma Cells
Source: PLoS One. 2012 Jan 17;7(1):e29021. doi: 10.1371/journal.pone.0029021 (PMC3260146; doi:10.1371/journal.pone.0029021)
Supplement: Table S3 — Top 20 enriched TF binding motifs in PR-binding sites in leiomyoma cells but not in breast cancer cells. (DOC) [file pone.0029021.s004.doc]

# Supplemental Table S3

Top 20 enriched TF binding motifs in PR-binding sites in leiomyoma cells but not in T47D breast cancer cells (based on a subset of the 100 most highly ranked TRANSFAC matrices for each cell type).

| **Factor name** | **Description** | **Motifs (TRANSFAC**  **matrix Id)** | **Z-value** | **p-value** |
| --- | --- | --- | --- | --- |
| AP-1 | Activating protein 1; Fos/Jun | V$AP1_01 | 99.979 | < 1.0e-323 |
|  |  | V$AP1_C | 98.060 | < 1.0e-323 |
|  |  | V$AP1_Q6_01 | 68.980 | < 1.0e-323 |
|  |  | V$AP1_Q6 | 62.082 | < 1.0e-323 |
|  |  | V$AP1_Q4_01 | 53.804 | < 1.0e-323 |
|  |  | V$AP1_Q2_01 | 52.675 | < 1.0e-323 |
|  |  | V$AP1_Q4 | 47.761 | < 1.0e-323 |
|  |  | V$AP1_Q2 | 38.001 | < 1.0e-323 |
| Bach1 | Transcription regulator protein BACH1; Basic- | V$MAF_Q6_01 | 58.803 | < 1.0e-323 |
|  | leucine zipper TF MafG | V$BACH1_01 | 43.138 | < 1.0e-323 |
| Fra-1 | FOS-like antigen 1; FOSL1 | V$FRA1_Q5 | 53.804 | < 1.0e-323 |
| HSF 1,2 | Heat shock factor 1 and 2 | V$HSF1_01 | 19.344 | 2.1e-079 |
|  |  | V$HSF2_01 | 11.038 | 1.3e-024 |
| Kid3 | Zinc finger KID3 | V$KID3_01 | -23.923 | 2.0e-122 |
| NeuroD | Neurogenic differentiation factor 1 | V$NEUROD_01 | 19.150 | 8.7e-078 |
| NF-E2 | Nuclear factor erythroid 2 p45; NFE2 | V$NFE2_01 | 46.094 | < 1.0e-323 |
|  |  | V$NRF2_Q4 | 35.447 | 5.3e-271 |
| PR | Progesterone receptor | V$PR_02 | 44.274 | < 1.0e-323 |
|  |  | V$PR_01 | 38.333 | < 1.0e-323 |
| TEF-1 | Transcriptional enhancer factor 1; Sph factor | V$TEF1_Q6 | 31.190 | 2.1e-209 |
|  |  | V$TEF_01 | 26.184 | 5.0e-147 |
| C/EBP α,β | CCAAT Enhancer Binding Protein α and β; | V$CEBPB_01 | 17.322 | 2.6e-063 |
|  | CEBPA; CEBPB | V$CEBP_Q2_01 | 16.299 | 7.7e-056 |
|  |  | V$CEBP_Q2 | 16.022 | 6.7e-054 |
|  |  | V$CEBPA_01 | 12.691 | 3.9e-033 |
| Cdx-1,2 | Caudal type homeobox transcription factor 1 and 2; | V$CDX1_01 | 14.799 | 1.0e-045 |
|  | CDX1; CDX3 |  |  |  |
| CREB | Cyclic AMP responsive element binding factor | V$TAXCREB_02 | 13.688 | 7.7e-039 |
| HOXC 10,11,12 | Ce-LPH1-binding factor; Hox-3.6; Hox-3.7; Hox-3.8 | V$HOXC12_01 | 17.481 | 1.7e-064 |
|  |  | V$HOXC11_01 | 16.564 | 9.8e-058 |
|  |  | V$HOXC10_01 | 16.250 | 1.7e-055 |
| MafB | V-maf Musculoaponeurotic fibrosarcoma oncogene | V$MAFB_01 | 15.888 | 5.7e-053 |
|  | homolog B (avian); MAFB |  |  |  |
| NF-AT1 | Nuclear factor of activated T-cells p; NF-AT1 | V$NFAT1_Q6 | 14.547 | 4.2e-044 |
| STAT 3,1 | Signal transducer and activator of transcription 3 and 1; | V$STAT3_03 | 15.275 | 8.0e-049 |
|  | STAT3; STAT1; STAT5a; STAT5b | V$STAT5B_01 | 13.488 | 1.2e-037 |
|  |  | V$STAT1_01 | 12.447 | 8.5e-032 |
|  |  | V$STAT_Q6 | 12.318 | 4.2e-031 |
|  |  | V$STAT3_01 | 11.305 | 6.6e-026 |
| CTF- 1,2,3,5,7 | CAAT-binding transcription factor 1, 2, 3, 5, and 7; | V$NF1_Q6_01 | 12.097 | 6.2e-030 |
|  | CTF-1; CTF-2; CTF-3; CTF-5; CTF-7 | V$NF1_Q6 | 12.024 | 1.5e-029 |
| AML1 | Acute myeloid leukemia 1; CBFA2; RUNX1 | V$AML1_Q4 | 11.394 | 2.4e-026 |
| CAR | Constitutive androstane receptor; MB67 | V$DR4_Q2 | -11.119 | 5.3e-025 |
| COUP-TF2 | Apolipoprotein repressor protein 1; ARP-1 | V$ARP1_01 | -10.844 | 1.1e-023 |
